# Visual Attention to Dynamic Emotional Faces in Adults on the Autism Spectrum

**DOI:** 10.1007/s10803-023-05979-8

**Published:** 2023-04-20

**Authors:** Sylwia Macinska, Shane Lindsay, Tjeerd Jellema

**Affiliations:** Department of Psychology, Faculty of Health Sciences, University of Hull, Cottingham Road Hull, Hull, HU6 7RX UK

**Keywords:** Eye-tracking, High-functioning autism, Autism spectrum, Dynamic facial expressions, Gaze, Perceptual history

## Abstract

Using eye-tracking, we studied allocation of attention to faces where the emotional expression and eye-gaze dynamically changed in an ecologically-valid manner. We tested typically-developed (TD) adults low or high in autistic-like traits (Experiment 1), and adults with high-functioning autism (HFA; Experiment 2). All groups fixated more on the eyes than on any of the other facial area, regardless of emotion and gaze direction, though the HFA group fixated less on the eyes and more on the nose than TD controls. The sequence of dynamic facial changes affected the groups similarly, with reduced attention to the eyes and increased attention to the mouth. The results suggest that dynamic emotional face scanning patterns are stereotypical and differ only modestly between TD and HFA adults.

During social interaction, people tend to look at each other’s faces and in particular at the eyes. This persistent attentional looking bias is in typically-developing (TD) individuals present from a very young age (e.g. Gliga & Csibra 2007), and is thought to be critical for the development of social and communicative skills (Johnson et al., 1991). Eye-tracking technologies have been instrumental in detecting and characterising spontaneous looking patterns, which are frequently used as a measure for where overt visual attention is directed (Guillon et al., 2014). Visual exploration of the face, as revealed by eye-tracking, is typically characterised by a triangular fixation pattern on eyes, nose, and mouth, but with the majority of attentional resources allocated towards the eyes (Walker-Smith et al., 1977). Individuals with Autism Spectrum Disorder (ASD; American Psychiatric Association, DSM-5, 2013) show aberrant visual social attention. Infants with ASD of about 6 months old are already less attentive to people, especially to their faces (Dawson et al., 1998), reflected by atypical face tracking behaviour (e.g. Ozonoff et al., 2010). It is often assumed that individuals with ASD pay less attention to the eyes and increased attention to the mouth and rest of the head compared to TD individuals. However, this dissociation of the targets of visual attention between TD and ASD, which could potentially determine the social and emotional information that can be gathered from faces in ASD, is not unanimously supported. Although quite a few studies reported reduced fixation on the eye region in ASD (e.g. Dalton et al., 2005; Jones et al., 2008; Klin et al., 2002; Nuske et al., 2014; Pelphrey et al., 2002; Spezio et al., 2007), others reported similar fixation patterns in TD and ASD (e.g. Bar-Haim et al., 2006; McPartland et al., 2011; Rutherford & Towns, 2008; Sawyer et al., 2012; Van der Geest et al., 2002; Van der Donck et al., 2021). Reviews and meta-analyses confirmed the uncertainty with respect to differences in face scanning patterns in TD and ASD (Chita-Tegmark, 2016; Falck -Ytter & Von Hofsten, 2011; Guillon et al., 2014).

As to explanations for these mixed results, Åsberg Johnels et al. (2017) suggested that the participant’s age may be a factor, with aberrant scanning patterns occurring especially in younger ASD participants, and normalisation of scanning of the eye region during adulthood. However, other factors related to the face stimuli and experimental procedures may also influence the scanning patterns, such as (i) the gaze direction (toward versus away from the observer), (ii) the facial expression, (iii) the static versus dynamic presentation mode of the faces, and the influence of the perceptual history when there is a dynamic sequence of expressions, and (iv) whether viewing of the stimuli was done in a spontaneous manner or during performance of a visual task.

*Gaze direction.* Although many eye-tracking studies manipulated the emotional facial expression, the influence of gaze direction on viewing patterns of these emotional facial expressions remained largely unexplored. This is surprising as it is well established that eye gaze can qualify the meaning of a facial expression; for example, direct gaze enhances the processing of approach-oriented emotions such as joy and anger, while averted gaze enhances the processing of avoidance-oriented emotions such as fear and sadness (Adams & Kleck, 2003, 2005; Hudson & Jellema, 2011). Further, direct gaze is detected faster and attracts longer visual attention than averted gaze (Senju & Hasegawa, 2005), and facial expressions of anger with gaze directed at the observer may lead to reduced viewing of the eye region (Sepeta et al., 2012). The hyper-arousal model assumes that reduced fixations at the eye region in ASD are caused by perception of the eyes as threatening and over-arousing (Dalton et al., 2005; Tanaka & Sung, 2016). While mutual gaze is intrinsically rewarding for TD individuals, individuals with ASD may fail to form such positive associations, and may even develop a negative response towards direct gaze, possibly due to overly high physiological arousal (Kliemann et al., 2010). Thus, studies presenting exclusively directed gaze may be biased towards finding an avoidance of the eye region in ASD.

*Facial expressions.* The emotion displayed on the face may influence attention allocation, with for example expressions of joy attracting relatively more attention to the mouth region (e.g. Eisenbarth & Alpers 2011). Importantly, face exploration may differ between TD and ASD individuals depending on type of expression, with some reports indicating that individuals with ASD avoid the eye region of specifically negative emotional expressions (Kliemann et al., 2010, 2012).

*Dynamic flow of expressions and perceptual history*. In the real-world, facial expressions seldom appear as a static image in isolation, but typically form part of a natural dynamic flow of facial expressions (cf. Sato et al., 2019; Calvo et al., 2018). Moreover, the dynamic flow of facial expressions is typically accompanied by dynamic changes in gaze direction. The scanning pattern of the final static expression that immediately follows such a fluid, dynamic, interplay of facial cues - which we describe here as the immediate perceptual history - may well be influenced and guided by this perceptual history. In particular because the perceptual history contains information about the emotional/mental state of mind of the agent, and may therefore qualify the perception and meaning of the subsequent static expression (Palumbo & Jellema, 2013). For example, the meaning and intensity of a smile that immediately follows an expression of anger may be perceived as more intense, due to greater contrast with the initial expression, than a smile without perceptual history (Palumbo & Jellema, 2013). Keeping track of the immediate facial perceptual history is thus socially important. Nevertheless, the majority of eye-tracking studies tested isolated static images, where the to-be-scanned expression is preceded by a fixation cross, rather than ecologically-valid stimuli (see Chita-Tegmark 2016, for a review). Just a few studies have specifically examined dynamic facial expressions (e.g. Åsberg Johnels et al., 2017; Pelphrey et al., 2002; Calvo et al., 2018). Importantly, anomalies in the perception of dynamic sequences of facial expressions have been reported in ASD, including atypical emotional anticipation of upcoming facial expressions (Palumbo et al., 2015, 2018).

*Spontaneous viewing.* It is important to differentiate between spontaneous, passive, viewing of emotional faces on the one hand and effortful viewing as when the observer performs an emotion recognition or visual rating task on the other. There are strong indications that during task performance a different attentional strategy is employed than during spontaneously observing the facial expressions, reflected in a different scanning pattern (Hunnius et al., 2011; Schurgin et al., 2014).

There are thus a number of outstanding questions, concerning the way in which spontaneous face scanning behaviour on the autism spectrum is influenced by gaze direction, by facial expression, by presentation mode and by the immediate perceptual history of the face.

## The Current Study

The study consists of two experiments in which eye-scanning behaviour of dynamic sequences of human faces is recorded during spontaneous viewing. Both the facial expression and gaze direction of the on-screen presented faces were dynamically manipulated in short video clips (2s). The clips started and ended with ‘opposite’ static emotional expressions (joy or anger) and ‘opposite’ gaze direction (toward or away), while during the middle part the expressions and gaze directions morphed into each other in a smooth, natural way. The influence of the perceptual history was assessed by comparing each of the four emotion-gaze combinations presented in the initial static phase of the clips with the identical combination presented in the final phase in other clips. While the initial phase can be seen as an isolated event without a perceptual history (i.e. preceded by the fixation cross), the final phase is informed by the immediate preceding history regarding the agent’s emotional state of mind (i.e. changes in the agent’s facial expression and gaze direction). The sequences were observed under free-viewing conditions; we deliberately did not instruct participants to perform a face-related task during scanning to avoid any contamination of the scanning pattern by task strategy.

In Experiment 1, we examined TD individuals with either low or high scores on the Autism Quotient (AQ; Baron-Cohen et al., 2001). The AQ measures the level of autistic-like traits in the general population, which traits have been reported to affect social perception (e.g. Chakrabarti et al., 2009; Hudson et al., 2012). For example, aberrant fixation behaviour toward faces with direct gaze was observed in children and adolescents scoring high on the AQ (e.g. Åsberg Johnels et al., 2017; Chen & Yoon, 2011), which mirrors results found in ASD. In Experiment 2, we examined individuals with high-functioning autism (HFA), and a matched control group of TD individuals, using the same paradigm.

The main aim was to explore which factors (gaze direction, emotion type, perceptual history) influence the scanning patterns of emotional faces in individuals with TD and HFA. These factors are still very much understudied in eye-tracking studies, which is especially true for the perceptual history. It has been argued that the immediate perceptual history - concerning changes in the facial expression and gaze direction – gives rise to involuntary emotional anticipation effects in TD, but not in ASD, individuals (e.g. Palumbo et al., 2015). We further aimed to determine whether any atypicalities in the HFA group may extend into the TD population, by examining TD groups who scored either low or high on the AQ. The presence of such atypicalities in the high AQ group, but not in the low AQ group, would support the notion of an underlying autism continuum with respect to visual attention allocation to faces (cf. Baron-Cohen et al., 2001).

Although the four groups - TD with low AQ, TD with high AQ, HFA, and matched TD - were administered the same experimental paradigm, we analysed the data from the first two groups, and the last two groups, separately (Experiments 1 and 2, respectively), to reflect the two main research questions. In Experiment 1, we investigated whether amongst TD individuals, a difference in face scanning behaviour may exist in relation to their AQ scores, while in Experiment 2 we investigated whether individuals with HFA show anomalous face scanning as compared to matched TD controls. To further explore a possible underlying continuum on the autism spectrum with respect to allocating visual attention to facial features, an final overall exploratory analysis was performed in which the TD group with low AQ, the TD group with high AQ, and the HFA group were included.

## Experiment 1: TD Individuals with Low and High AQ Scores

### Methods

#### Participants

Forty-four undergraduate TD students from the University of Hull participated in the experiment in exchange for course credits. Data of five participants was excluded due to technical errors, leaving a final sample of 39 individuals. This sample size, with 20 and 19 participants in the low and high AQ groups, respectively (see below), is representative of the studies in this field. The meta-analysis by Chita-Tekmark (2016), which included 64 studies that compared TD and ASD groups in their face scanning behaviour, showed that on average 19 TD individuals (*SD* = 8.2) and 16 ASD individuals (*SD* = 6.9) participated in these experiments. These studies revealed a mean effect size (Cohen’s d) for the difference in fixation times of the eye region of 0.43 (small to medium size). All participants had (corrected-to) normal vision, and none reported to be suffering from any psychological or (neuro-)developmental conditions. They completed an online Autism Quotient questionnaire (Baron-Cohen et al., 2001; https://psychology-tools.com/test/autism-spectrum-quotient). The AQ is a self-administered 50-item questionnaire, designed to measure the degree to which an adult with normal intelligence possesses autistic-like traits. Their AQ scores ranged from 6 to 30, with a median score of 17, resulting in 20 participants (11 females, 9 males) in the low AQ group (AQ scores < 17; *M* = 13.2, *SD* = 2.6) and 19 participants (13 females, 6 males) in the high AQ group (AQ scores > 16; *M* = 21.8, *SD* = 4.5). The two AQ groups did not differ in age (low AQ: *M* = 19.3, *SD* = 2.0; high AQ: *M* = 19.9, *SD* = 2.6; *t*(37) = 0.871, *p* = 0.39) nor in gender ratio (*X*_*2*_(1, *N* = 39) = 0.74, *p* = 0.39).

#### Stimuli

Eight photographs of actors were selected from the Warsaw set of Emotional Facial Expressions Pictures (WSEFEP; Olszanowski et al., 2015), depicting four different models (frontal views only, two males, RA and MK, and two females, SS and MS) with facial expressions of joy and anger. The models were selected on the basis of high rates of agreement for expressions of joy and anger (Olszanowski et al., 2015), and for ample eye size ensuring manipulation of gaze direction would be clearly visible. The two photographs of each model (joy and anger) were interpolated with 18 images, to create dynamic face stimuli displaying a natural facial movement (Perrett et al., 1994; Sqirlz Morph software).

**Fig. 1:**
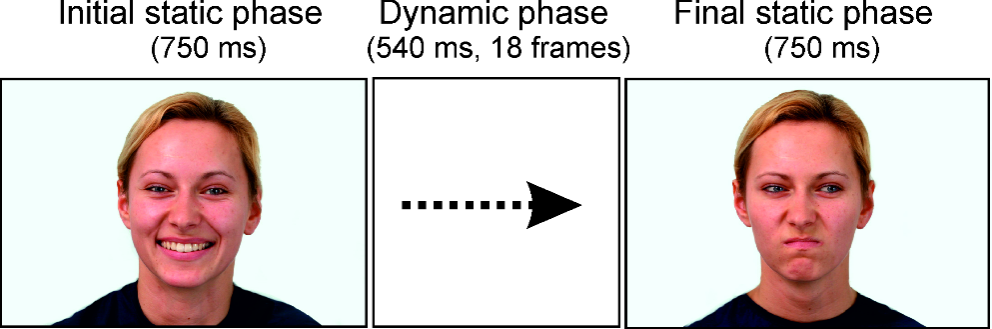
Schematic representation of a video-clip. The Happy-Direct to Angry-Averted condition is depicted.

Each video started with a static frame presented for 750 ms, followed by the dynamic sequence consisting of the 18 interpolated frames of 30 ms each (540 ms), and ended with a static frame of 750 ms duration (total duration 2040 ms, Fig. 1). Two types of videos were created. In the first type the initial frame depicted the agent with an expression of joy and with their gaze directed towards the observer. During the dynamic phase (540 ms), the expression of joy gradually morphed into anger with maximal anger reached at the end of the dynamic phase, while the directed gaze gradually changed into averted gaze reaching a 30° angle away from the observer at the end of the dynamic phase. The second type of video started with an expression of anger and gaze directed at the observer, with the expression of joy gradually changing into anger, and the directed gaze gradually changing into averted gaze (30° angle) away from the observer, during the dynamic phase. Both videos were also played backwards resulting in four conditions. Thus, half of the trials began with a character displaying joy (in 50% of these trials gaze was directed at, and in the other 50% directed away from, the observer), while the other half of the trials began with an expression of anger (in 50% directed at observer, and in 50% averted away).

#### Procedure

Participants were seated at 70 cm in front of a computer screen in a darkened room. Their eye movements were recorded using a head-mounted EyeLink 1000 tracker (SR Research Ltd.), sampling at 1000 Hz, with the default 9-point EyeLink calibration. Participants could only proceed when the average fixation error was < 0.5°, and when no point produced a fixation error > 1°. Calibration was then validated in a 9-point test, and calibration was repeated when these criteria were not met in the validation. Viewing was binocular but the camera recorded the eye movements from the right eye only. A chin rest was used to stabilize the participant’s head and to ensure the distance from the monitor remained fixed. Participants were instructed that the aim of the study was to assess their perception of faces and that they simply should observe the videos. Other than that, no task was given.

Each trial started with a fixation cross presented at the centre of the screen for 1500 ms, directly followed by the video-clip. Participants viewed a total of 64 videos (4 conditions x 4 characters x 4 repetitions). After completion of the experiment participants completed the online AQ questionnaire.

#### Fixation Time Analysis

**Fig. 2:**
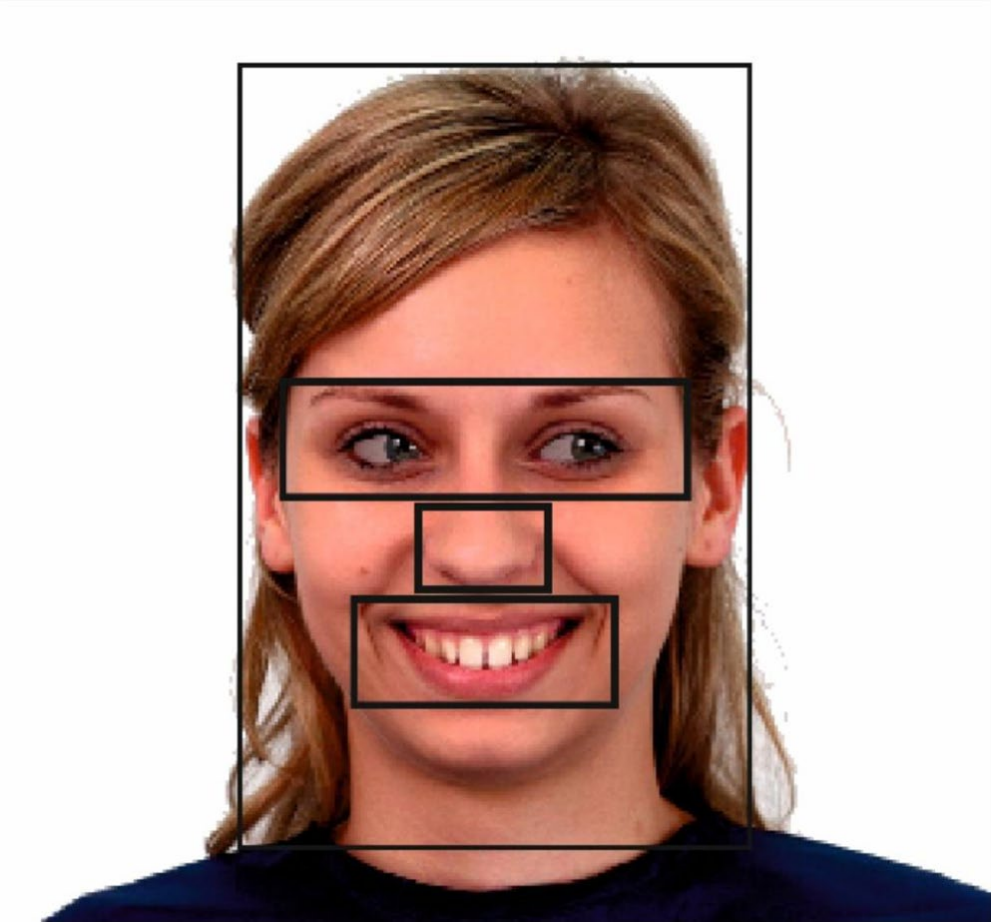
The areas of interest (AOIs). From small to large, the rectangles indicate the nose, mouth, eyes, rest of head, and background areas.

For each model, four rectangular areas of interests (AOI) covering eyes, mouth, nose, and rest of head, were defined prior to data collection (Fig. 2). These AOIs were equivalent in size across the models and emotions. A fifth AOI consisted of the screen area outside the head area, and also included off-screen fixations. Fixations were predefined as consecutive eye gaze positions focused within an area of one visual degree for a period of 100 ms or more (Manor & Gordon, 2003). Gaze fixations below this threshold were not included in the analysis. The total fixation time made to each AOI was calculated for each trial. This was expressed in our analysis as the mean percentage of total fixation time.

## Results

As fixations shorter than 100 ms and blinks had been removed from the data, we first checked whether the AQ groups differed in their overall fixation time on all AOIs, as these short fixations and blinks may not have been equally distributed over the two groups. Hereto the average of total fixation time as a function of total stimulus presentation time (2040 ms) was calculated for each participant. The groups did not differ in fixation time (*t*(37) = 0.71, *p* = 0.48), with the low AQ group fixating at (and outside) the screen for 79% of the presentation time and the high AQ group for 80%. We conducted a 5 × 2 × 2 × 2 × 2 repeated measures ANOVA on the percentages of time spend fixating in each of the five AOIs. The within-subject factors were AOI (eyes, mouth, nose, rest of head, background), Emotion (joy, anger), Gaze direction (direct, averted), and Phase (initial, final), and the between-subject factor was Group (low AQ, high AQ). Mauchly’s test indicated that the assumption of sphericity had been violated, therefore degrees of freedom were corrected using Greenhouse-Geisser estimates.

The main effect of the factor Group was non-significant [*F*(1,37) = 4.07, *p* = 0.051, ηp^2^ = 0.099].

*Areas of interest (AOI).* The main effect of AOI was significant [*F*(1.30, 47.9) = 147.1, *p* < 0.001, ηp^2^ = 0.80]. Pairwise comparisons revealed a significantly longer looking time at the Eye region compared to all other AOIs (all *p*’s < 0.001). Overall, the Eye region was looked at for 65% of the time (*SD* = 21.8) (Nose, 10.1%; Mouth, 9.7%; Rest of head, 13.6%; Background, 1.6%). Fixation times at the Mouth and Nose regions did not differ (*p* = 0.83). The allocation of attention to specific AOIs was influenced by the agent’s facial expression [AOI by Emotion interaction [*F*(2.07,76.5) = 15.5, *p* < 0.001, ηp^2^ = 0.30] For expressions of joy, the fixation time on the Mouth was significantly higher (*p* < 0.001) and the fixation time on the Nose significantly smaller (*p* = 0.008), than for expressions of anger. Fixation times on the Eye region were not affected by facial expression (*p* = 0.54). The gaze direction displayed by the agent did not affect allocation of attention to the AOIs, AOI by Gaze interaction [*F*(3.1, 115) = 1.48, *p* = 0.21, ηp^2^ = 0.038]. The separation of the participants in low and high AQ groups did also not have a significant impact on attention allocation to the AOIs [AOI by Group interaction, *F*(1.30,47.9) = 2.99, *p* = 0.080, ηp^2^ = 0.075], with the low AQ group fixating the eye region for 58.8% and the high AQ group for 71.5%. The remaining interactions of AOI with Emotion, Gaze and Group were non-significant.

*Initial vs. final phase.* The factor Phase represented the influence of the perceptual history on scanning behaviour, which was achieved by comparing identical faces (same expression and gaze direction) presented either in the initial or in the final phase of the video-clips. The main effect of Phase [*F*(1,37) = 0.53, *p* = 0.47, ηp^2^ = 0.014] and the Phase by Group interaction [*F*(1,37) = 0.81, *p* = 0.37, ηp^2^ = 0.021] were both non-significant, reflecting that the immediate perceptual history did not influence the face scanning of the low and high AQ groups. The remaining 2-, 3-, and 4-way interactions of Phase with Group, Emotion and Gaze were all non-significant. There was, however, a highly significant interaction of Phase with AOI [*F*(2.2,81.7) = 28.6, *p* < 0.001, ηp^2^ = 0.44). This interaction reflected a drop in fixation time on the Eye region from initial to final phase (*p* < 0.001), accompanied by an increase in fixation time on the Mouth region from initial to final phase (*p* < 0.001). The Phase by AOI by Emotion interaction was also significant [*F*(2.4, 87.5) = 6.56, *p* < 0.001, ηp^2^ = 0.15], which reflected that the increase in fixation time to the Mouth region in the final phase occurred significantly more for facial expressions of joy than for those of anger [Emotion by Phase interaction for fixation time at the mouth region, *F*(1, 38) = 27.4, *p* < 0.001, ηp^2^ = 0.42]. The AQ groups did not differ in the extent to which they showed this increase in fixation time at the Mouth region for emotions of joy [Phase by AOI by Emotion by Group, *F*(2.7,97.0) = 0.81, *p* = 0.52, ηp^2^ = 0.021].

The main effects of Emotion and Gaze were both non-significant, as were the Emotion by Gaze interaction [*F*(1,37) = 0.54, *p* = 0.50, ηp^2^ = 0.014], and their interactions with Group [Emotion by Group, *F*(1,37) = 0.45, *p* = 0.51, ηp^2^ = 0.012; Gaze by Group, *F*(1,37) = 0.33, *p* = 0.57, ηp^2^ = 0.009].

**Fig. 3:**
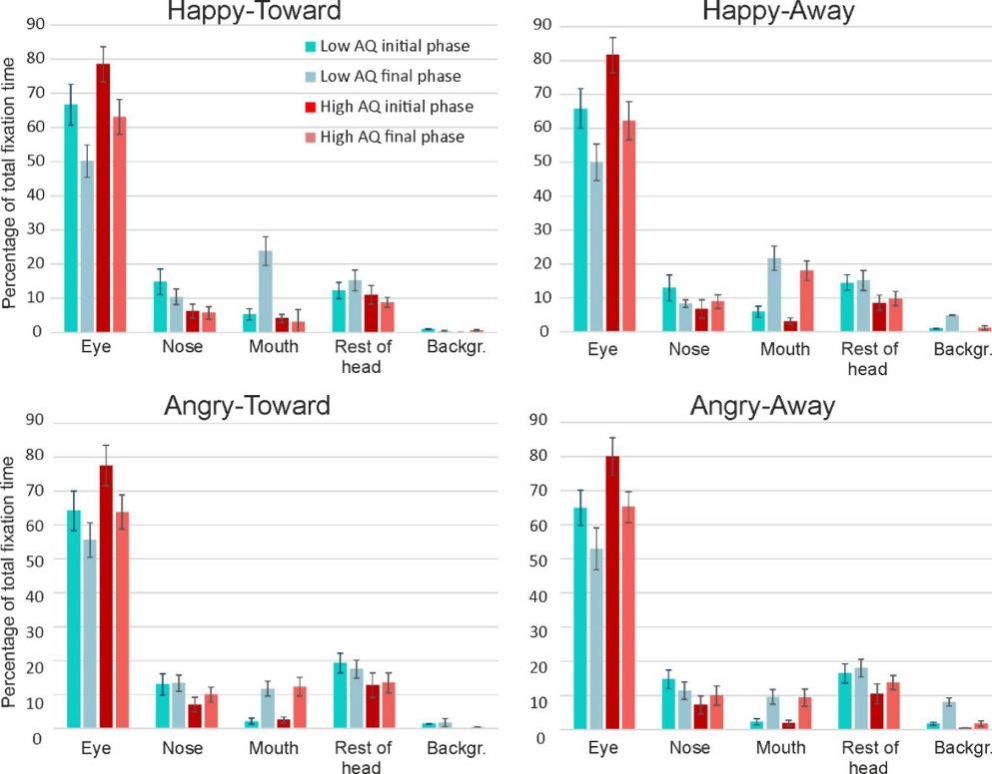
The average percentages of time that individuals in the low and high AQ groups spend fixating in the AOIs during presentations of identical face stimuli during the initial and last phases of the clips, for each of the four emotion-gaze combinations. Error bars indicate s.e.m.

**Fig. 4:**
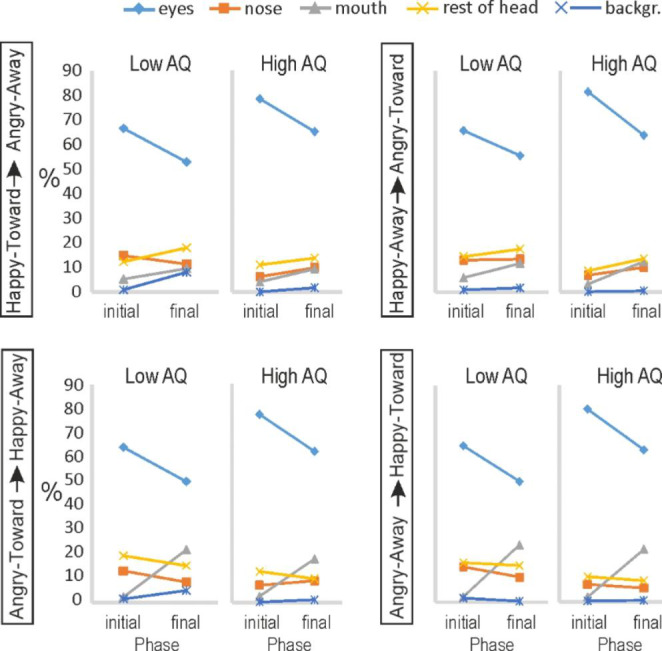
Fixations of TD participants in the low and high AQ groups in each of the four video-clip types. Mean percentage of looking time at the six AOIs are shown during the initial and final frames in the four video-clips.

To illustrate the shifts in attention from the initial to the final faces within clips, the data presented in Fig. 3 is shown in Fig. 4 per video-clip type (clip 1: Happy-Toward to Angry-Away; clip 2: Happy-Away to Angry-Toward; clip 3: Angry-Toward to Happy-Away; clip 4: Angry-Away to Happy-Toward). Here, the initial and final phases represent opposite emotions (joy vs. anger) and opposite gaze direction (toward vs. averted).

### Main Findings Experiment 1

Overall, the low and high AQ groups showed similar fixation patterns, with both groups looking predominantly at the eye region. Even though in all four basic conditions (Happy-Toward, Happy-Away, Angry-Toward, Angry-Away) the high AQ group looked for a higher percentage of time at the eyes than the low AQ group, these percentages did not differ significantly. The allocation of fixation time across the AOIs was similarly influenced by the displayed facial expression in the two groups: for expressions of joy, the mouth region was fixated significantly more than for expressions of anger, while the nose region was fixated significantly less for expressions of joy compared to anger. Gaze direction did not influence the fixation distribution in either group. The immediate perceptual history did not affect face scanning behaviour in either group, except for a specific interaction with AOI and Emotion. That is, in both groups, the increase of fixations to the mouth area for expressions of joy, compared to anger, almost exclusively occurred in the final phase (i.e. following an immediate perceptual history).

## Experiment 2: HFA Individuals and Matched Controls

### Methods

The methodology and procedures for Experiment 2 were the same as for Experiment 1.

The two experiments only differed with respect to participant groups and the associated diagnostic procedures.

#### Participants

Eighteen undergraduate students with HFA at the University of Hull were recruited through University Disability Services. They received £15 for participating. One participant was excluded due to eye-tracking calibration issues, so that the final sample consisted of 17 participants (4 females, 13 males), with a mean age of 19.4 years (*SD* = 0.9). They all had previously received a diagnosis of HFA or Asperger’s syndrome from a clinical psychologist or psychiatrist. None reported co-occurring conditions (such as ADHD, anxiety, or depression). Diagnosis of HFA was confirmed using the ADOS (Autism Diagnostic Observation Schedule, module 4), administered by a qualified experimenter (SM). The ADOS is a semi-structured, standardized assessment designed for use with children and adults suspected of having ASD. Their mean ADOS score was 9.7 (*SD* = 2.2). Their mean total IQ score was 113.6 (*SD* = 7.9), as determined by a short version of the Wechsler Adult Intelligence Scale (WAIS-IV; Wechsler, 1997). Their mean AQ score was 34.2 (*SD* = 7.8, range 18 to 46). All had (corrected-to) normal vision.

The control group, selected to match the HFA participants in age, sex and IQ, consisted of 19 undergraduate TD students from the University of Hull (6 females, 13 males, age: *M* = 19.4 years, *SD* = 1.6). All participated in exchange for course credits. Their mean total IQ score, assessed with the short version of the Wechsler Adult Intelligence Scale (WAIS-IV; Wechsler, 1997), was 114.5 (*SD* = 6.4). Their mean AQ score was 16.7 (*SD* = 5.1, range 10 to 28). All participants had (corrected-to) normal vision, and none reported to be suffering from any psychological or (neuro-)developmental conditions. The HFA and TD groups did not differ significantly in age (*t*(34) = 0.035, *p* = 0.97), gender ratio (*X*_*2*_(1, N = 36) = 0.020, *p* = 0.89) or IQ (*t*(34) = 0.112, *p* = 0.91). The AQ scores were higher in the HFA group (*t*(34) = 8.0, *p* < 0.001). It was essential to include a matched TD control group in Experiment 2, rather than including the combined low and high AQ groups for that purpose, due to differences in gender ratios (i.e. the HFA group contained significantly more male participants than the low/high AQ group). Moreover, no IQ scores had been obtained from the low/high AQ participants that took part in experiment 1.

## Results

After removal of blinks and of fixations shorter than 100 ms, the HFA and matched TD (mTD) groups did not differ significantly in their overall fixation time [*t*(34) = 1.25, *p* = 0.22], with the HFA group looking at (and outside) the screen for 77% of the presentation time and the matched TD group for 79%.

Similar to Experiment 1, a 5 × 2 × 2 × 2 × 2 repeated measures ANOVA was conducted, with as within-subject factors AOI (eyes, mouth, nose, rest of head, background), Emotion (joy, anger), Gaze direction (direct, averted), and Phase (initial, final), while the between-subjects factor Group consisted of the matched TD and HFA groups. The dependent variable was again the average percentage of time spend fixating in each of the five AOIs as a proportion of total fixation time per trial. Mauchly’s test indicated that the assumption of sphericity had been violated, therefore degrees of freedom were corrected using Greenhouse-Geisser estimates.

The main effect of the factor Group was non-significant [*F*(1,34) = 0.09, *p* = 0.76, ηp^2^ = 0.003].

*Areas of interest (AOI).* The main effect of AOI was significant [*F*(1.4,48.1) = 88.1, *p* < 0.001, ηp^2^ = 0.72]. In contrast to similar fixation patterns of the low and high AQ groups, the mTD and HFA groups allocated their attention differently to the AOIs [AOI by Group interaction, *F*(1.4,44.9) = 5.0, *p* < 0.001, ηp^2^ = 0.13]. Although each group fixated mostly on the Eye region [eyes vs. all other AOIs: for both mTD and HFA, all *p*s < 0.001], follow-up independent t-tests revealed that the HFA group fixated less on the Eyes, and more on the Nose and Rest of head areas, compared to the mTD group [Eyes region: HFA, 43.6%, *SD* = 21.9; mTD, 64.8%, *SD* = 19.8; *t*(34) = 3.1, *p* = 0.004; Nose region: HFA, 17.7%, *SD* = 9.1; mTD, 9.8%, *SD* = 7.5; *t*(34) = -2.9, *p* = 0.007; Rest of head region: HFA, 21.7%, *SD* = 7.9; mTD, 13.3%, SD = 8.4; *t*(34) = -3.2, *p* = 0.003; Bonferroni corrected α = 0.01]. The two groups did not differ with respect to the Mouth region [HFA, 11.4%, *SD* = 10.6; mTD, 9.7%, *SD* = 6.3; *t*(34) = -0.58, *p* = 0.57] nor the Background region [HFA, 5.6%, *SD* = 6.4; mTD, 2.4%, *SD* = 5.8; *t*(34) = -1.7, *p* = 0.10]. The AOI by Group by Emotion interaction was also significant [*F*(2.2,76.1) = 5.7, *p* < 0.001, ηp^2^ = 0.15]. However, the fixation time at the Eye region was not significantly affected by the facial expression in either the mTD or HFA group [HFA: joy, *M* = 45.0%, *SD* = 23.3; anger, *M* = 42.2%, *SD* = 20.7; *t*(16) = 2.0, *p* = 0.064; mTD: joy, *M* = 62.8%, *SD* = 20.3; anger, *M* = 66.8%, SD = 18.8; *t*(18) = -2.3, *p* = 0.036; Bonferroni corrected = 0.01]. The significant AOI by Group by Emotion interaction was driven by fixations at the Mouth region, with a significantly larger fixation time in the mTD group for expressions of joy compared to anger, but no such effect in the HFA group [mTD: joy, *M* = 13.4%, *SD* = 9.5; anger, *M* = 6.0%, *SD* = 5.1; *t*(18) = 4.4, *p* < 0.001; HFA: joy, *M* = 12.3%, *SD* = 11.7; anger, *M* = 10.6%, *SD* = 9.9; *t*(16) = 0.15, *p* = 0.14]. Facial expression did not affect fixation times at any of the remaining AOIs in either group. The factors Gaze and Phase did not further qualify this interaction.

*Initial vs. final phase.* The main effect of Phase [*F*(1,34) = 0.18, *p* = 0.68, ηp^2^ = 0.005] and the Phase by Group interaction [*F*(1,34) = 2.2, *p* = 0.15, ηp^2^ = 0.061] were both non-significant, reflecting that the face scanning of the HFA and mTD groups was not differentially affected by the immediate perceptual history. The remaining interactions of Phase with Group, Emotion and Gaze were all non-significant. However, the interaction of Phase by AOI was significant [*F*(2.7,90.1) = 21.8, *p* < 0.001, np^2^ = 0.39]. This interaction reflected a drop in fixation time from initial to final phase in the Eyes region (*p* < 0.001), and increases in fixation time from initial to final phase in the Mouth region (*p* < 0.001) and in the Rest of head region (*p* = 0.005). For the Nose and Background regions the differences between initial and final phases did not reach significance. These effects were further qualified by the facial expression [Phase by AOI by Emotion interaction, (*F*(2.7,92.7) = 5.7, *p* = 0.001, np^2^ = 0.15]. That is, the increase in fixation time to the mouth region from initial to final phase was significantly larger for facial expressions of joy than for those of anger (*p* < 0.001). The decrease in fixation time to the Eye region was not significantly affected by the facial expression (*p* = 0.08), nor was the increase in the final phase for the Rest of head region affected by facial expression (*p* = 0.89). The HFA and mTD groups did not differ in the extent to which they showed these effects [Phase by AOI by Emotion by Group, *F*(2.7,95.7) = 2.0, *p* = 0.11, np^2^ = 0.054].

*Emotion and Gaze.* The main effects of Emotion and Gaze were non-significant [Emotion, *F*(1,34) = 2.11, *p* = 0.16, ηp^2^ = 0.058; Gaze, *F*(1,34) = 0.32, *p* = 0.57, ηp^2^ = 0.009], the Emotion by Gaze interaction was also non-significant. Importantly, the interactions of each with Group were non-significant [Emotion by Group, *F*(1,34) = 2.11, *p* = 0.16, ηp^2^ = 0.058; Gaze by Group, *F*(1,34) = 0.32, *p* = 0.57, ηp^2^ = 0.009].

**Fig. 5:**
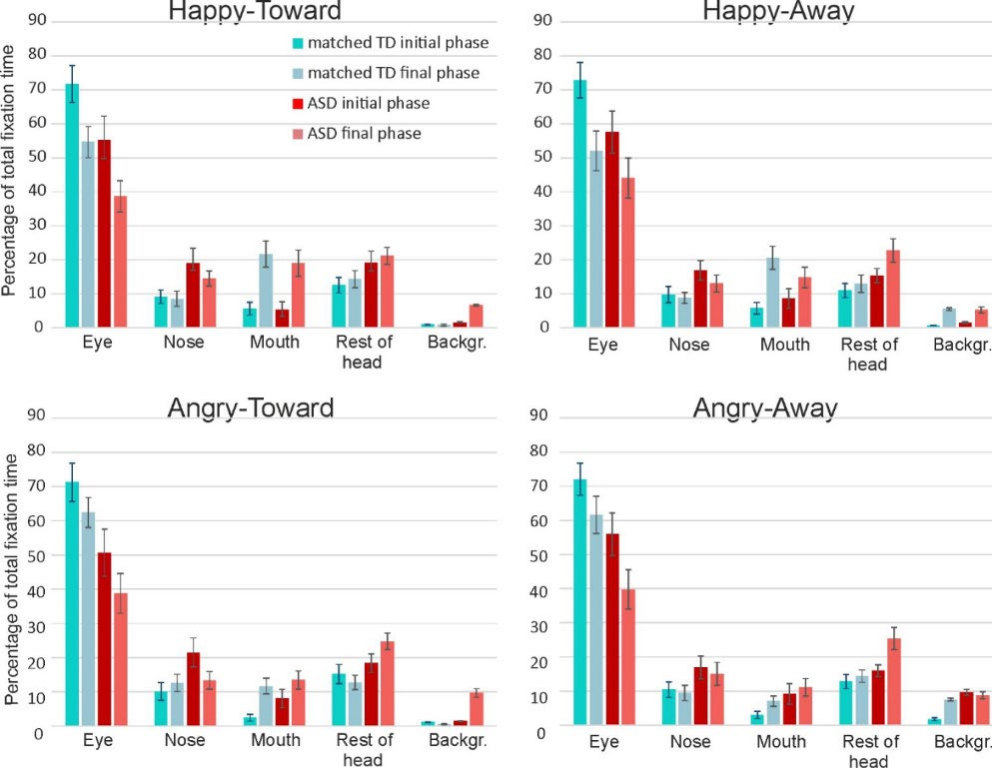
The average percentages of time that individuals in the mTD and HFA spend fixating in the AOIs during the initial and last phases of the video-clips, for each of the four emotion-gaze combinations. Error bars indicate s.e.m.

**Fig. 6:**
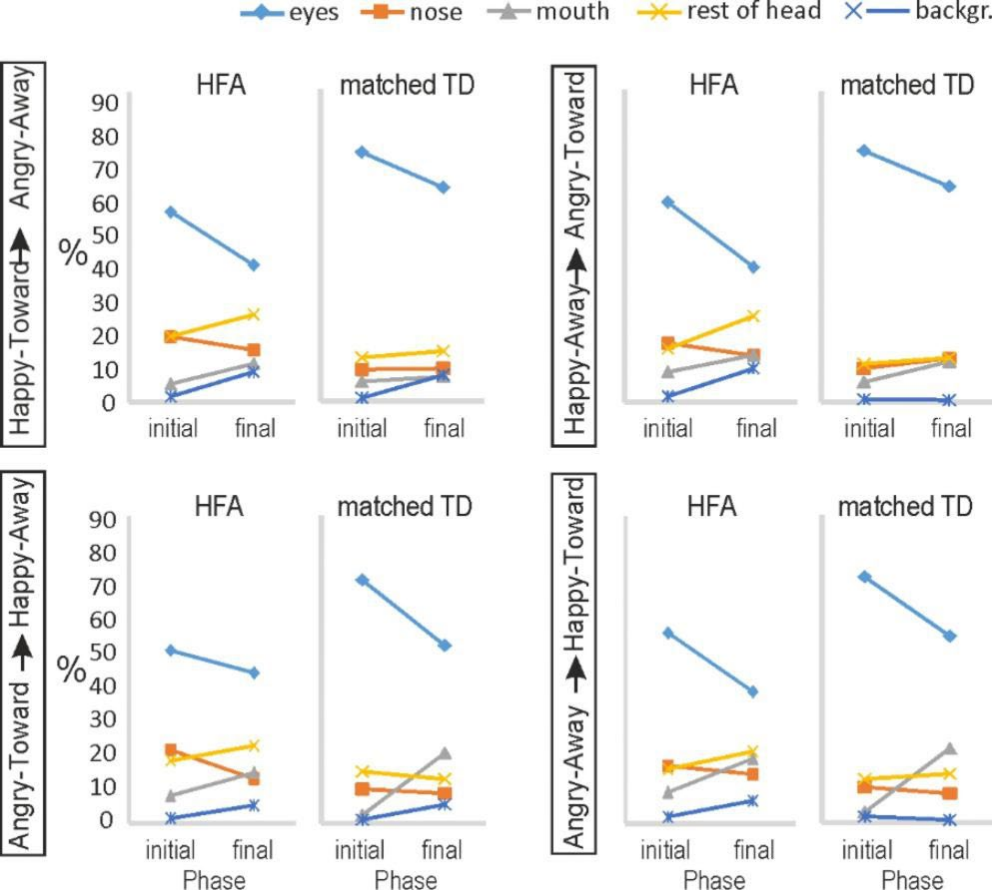
Fixation time of participants with HFA and matched TD controls. Mean percentage of fixation time at each of the six areas of interest are shown during the initial and final frames in the four types of video clips.

To help illustrate better the shifts in attention from the initial to the final faces within clips, the data presented in Fig. 5 is shown again in Fig. 6 per video-clip type (clip 1: Happy-toward to Angry-away; clip 2: Happy-away to Angry-toward; clip 3: Angry-toward to Happy-away; clip 4: Angry-away to Happy-toward). In Fig. 6 the initial and final phases thus represent opposite emotions (joy vs. anger) and opposite gaze direction (toward vs. averted).

## Main Findings Experiment 2

Experiment 2 revealed that the Eye region attracted most attention in both the mTD and HFA groups, as compared to all other AOIs. However, the HFA group fixated significantly less on the Eyes region than the mTD group, which effect was not modulated by the agent’s facial expression or gaze direction in either group. Conversely, the HFA group fixated significantly more on the Nose and Rest of the head areas than the mTD group, which was again not modulated by the agent’s facial expression and gaze direction in either group. Facial expression did, however, significantly affect fixation time at the Mouth region in the mTD group, with more fixation at the Mouth region for expressions of joy than for expressions of anger. For the HFA group, fixation time at the mouth was not influenced by the agent’s facial expression.

The face scanning of the HFA and mTD groups was not differentially affected by the immediate perceptual history. In both groups, there was a marked drop in fixation time to the Eyes region and an increase in fixation time to the Mouth region and the Rest of head region, from the initial to the final phase. Further, in both groups, the increase of fixation time to the mouth in the final phase was larger for expressions of joy than for anger, similar to what was found in Experiment 1.

### Comparison of the HFA Group with the Low\High AQ Groups

To investigate a possible autism spectrum continuum with respect to the allocation of attention to faces, the TD groups with low and high AQ scores from Experiment 1, and the HFA group from Experiment 2, were included in an exploratory overall 5 × 2 × 2 × 2 × 3 ANOVA to test this hypothesis. As in Experiments 1 and 2, the within-subject factors were AOI (eyes, mouth, nose, rest of head, background), Emotion (joy, anger), Gaze direction (direct, averted), and Phase (initial, final), while the between-subjects factor Group had 3 levels (TD low AQ, TD high AQ, HFA). Mauchly’s test indicated that the assumption of sphericity had been violated, therefore degrees of freedom were corrected using Greenhouse-Geisser estimates. The main effect of Group was not significant [*F*(2,53) = 2.17, *p* = 0.125, ηp^2^ = 0.076]. Of all interactions with Group, only the interaction with AOI was significant [*F*(2.76,73.2) = 4.52, *p* = 0.007, ηp^2^ = 0.15]. This interaction reflected significant differences in percentage of looking time between the HFA and High AQ group for all AOIs, except for the mouth region, whereas no significant differences were found between the HFA and Low AQ groups for any of the AOIs. Independent sample t tests for the HFA vs. High AQ comparison in each of the five AOIs were as follows (Bonferroni corrected α = 0.01): The HFA group looked less at the eyes (HFA, M = 43.6%; High AQ, M = 71.5%; *t*(34) = 3.9, *p* < 0.001), and looked more at the nose (HFA, M = 17.7%; High AQ, M = 7.7%; *t*(34) = 3.4, *p* = 0.002), more at the rest of the head (HFA, M = 21.6%; High AQ, M = 11.1%; *t*(34) = 3.5, *p* = 0.001) and more at the background (HFA, M = 5.6%; High AQ, M = 0.56%; *t*(34) = 3.4, *p* = 0.005). No difference was found for the mouth area (HFA, M = 11.4%; High AQ, M = 9.1%; *t*(34) = 0.77, *p* = 0.45).

## General Discussion

The aim of the current experiments was to determine the allocation of attention across diagnostic parts of emotional facial expressions, and how this allocation might change over time due to systematic changes in the facial expression and gaze direction of the face. The participants consisted of TD individuals with either low or high levels of autistic-like traits and individuals with HFA, so as to cover a large part of the autism spectrum. Attention was measured by the time spent viewing predetermined facial regions.

Overall, the four groups tested in this study (TD low AQ, TD high AQ, HFA and matched-TD) showed a fairly similar scanning pattern of the dynamic facial expressions. All groups fixated significantly more on the eyes than on any of the other facial areas, regardless of the displayed emotion or gaze direction. Gaze direction did not significantly influence the scanning pattern in any of the groups. The four groups also did not differ in their susceptibility to the immediate facial perceptual history. For all groups, the eyes of faces presented directly following the fixation cross (i.e. without perceptual history) were looked at more than the eyes of identical faces that were preceded by a dynamic facial perceptual history. For the mouth, the reversed effect was found in all four groups, that is an increase in looking time following the perceptual history as compared to without a perceptual history. However, there were also some marked differences. Compared to the mTD group, the HFA group spend less of their looking time in the eye region, and more in the nose region. Below we discuss the findings in more detail.

*Eyes.* The eyes attracted most attentional resources, compared to all other facial regions, in all groups and in all conditions. It has been argued that the eye region carries most information about the emotion, gender and identity (e.g. Peterson & Eckstein 2011). Therefore, it may be beneficial to explore this region first and only then pay more attention to the rest of the head. This may explain why the eyes at the start of the clips received more attention than identical eyes presented at the end of the clips. The predominant attention to the eyes was not influenced by the emotional expression and gaze direction of the face. With respect to the TD individuals, the combined low and high AQ groups spent 65.0% of their time attending the eyes (averaged over initial and final phases), while the matched TD group looked for 64.9% of their time at the eyes, which is in line with previous studies (e.g. Eisenbarth & Alpers 2011). The HFA group, however, looked for 43.6% of their time at the eyes, which was significantly less than the mTD group (64.8%). With respect to allocating attention to the eyes in ASD, the literature offers conflicting findings. Reduced attention towards the eye region in ASD has been found some studies (Chawarska & Shic, 2009; Dalton et al., 2005; Pelphrey et al., 2002; Spezio et al., 2007), but not by others (Bar-Haim et al., 2006; McPartland et al., 2011; Sawyer et al., 2012; Sepeta et al., 2012; Van Der Geest et al., 2002). This suggests the role of social attention is ASD is complicated and may vary depending on the context, task requirements, age and symptom severity (cf. Åsberg Johnels, 2017). It should be noted that in the current study viewing of the faces was spontaneous (no task requirements) and exclusively adult individuals with high-functioning autism were included. Aberrant eye viewing is mostly reported in children and adolescent with ASD, with the suggestion that adults may rely more on compensatory mechanisms (e.g. Åsberg Johnels, 2017). A reduction in attending to the eyes could potentially affect the amount of social information that is available to navigate the social world, contributing to social difficulties observed in ASD (APA, 2013).

*Emotional facial expression*. The emotional facial expression had a limited impact on scanning patterns. The only way in which it significantly influenced scanning was in relation to the mouth, which area was scanned more extensively for expressions of joy than for anger. The groups did not differ in this respect. This is in line with previous reports (Eisenbarth & Alpers, 2011; Schurgin et al., 2014; Calvo & Nummenmaa, 2008; Calvo et al., 2018) and is presumably due to the importance of the smile in the identification of joy (Schurgin et al., 2014). Some earlier studies also noted an increased attention to the eyes for expressions of anger (Schurgin et al., 2014; Smith et al., 2005; Calvo & Nummenmaa, 2008; Calvo et al., 2018), though others did not find this (De Wit et al., 2008; Eisenbarth & Alpers, 2011; Green et al., 2003; Hunnius et al., 2011). The current study found no support for increased attention to the eyes for expressions of anger. The provided viewing instructions may have contributed to these conflicting results, studies that failed to demonstrate it typically employed free-viewing, as did our study. During free-viewing, implicit strategies are at work of which the participant may not even be aware.

*Gaze.* The manipulation of the gaze direction did not significantly influence the scanning pattern in any of the groups. The hyperarousal model claims that eye-avoidance behaviour in ASD is caused by perception of the eyes directed at the observer as threatening and over-arousing (Dalton et al., 2005; Kliemann et al., 2010, 2012; Kylliäinen & Hietanen, 2006; Tanaka & Sung, 2016). Surprisingly, only a handful of studies directly compared the distribution of attention toward faces with direct versus averted gaze in ASD, yielding mixed findings. Some found increased looking times towards direct gaze in comparison to averted eyes in both TD and ASD individuals (Louwerse et al., 2013; Vivanti et al., 2011), but others found no differences in either group (Hernandez et al., 2009; Kaartinen et al., 2012; Nuske et al., 2015; Sepeta et al., 2012). One study found longer duration towards direct versus averted gaze in the TD group, but not in ASD (Vivanti & Dissanayake, 2014). There does not seem to emerge a clear pattern in scanning response to direct versus averted gaze in ASD.

*Perceptual history*. Comparing the scanning patterns for specific emotion/gaze combinations during the initial phase (750ms; directly following the fixation cross, thus no perceptual history) with those during the final static phase (750ms, with perceptual history) allowed to assess the influence on scanning patterns of the immediate perceptual history related to the agent’s changing emotional state of mind. The perceptual history influenced the scanning patterns equally in the four groups: in the last phase there was a decrease in scanning of the eyes and an increase in scanning of the mouth, compared to the initial phase, in all groups. Over the course of the video-clips, the morphological changes in the mouth were most pronounced given the emotions used, more so than the changes in the eye region or changes in gaze direction. This may have attracted attention to the mouth in the final phase. However, it happened predominantly when the expression changed from anger to joy, and not vice versa, while the extent of morphological change is the same. This suggests that it is not the extent of change in the shape of the mouth per se that triggered the additional attention to the mouth. Rather, the meaning or intensity of a smile that immediately follows an expression of anger, compared to that of a smile without perceptual history, may be responsible. A smile following an angry expression may be perceived as more intense due to greater contrast with the initial expression (cf. Palumbo & Jellema, 2013).

*AQ groups.* Our finding that TD individuals high in autistic-like traits focused on the diagnostic areas of the face to the same extent as TD individuals low in autistic-like traits contrasts with some previous studies (Chen & Yoon, 2011; Freeth et al., 2013; Vabalas & Freeth, 2016), who reported a reduced looking time at the eyes in high AQ individuals. Such a reduced scanning of the eyes in the high AQ group would fit in with the notion of an autistic traits continuum encompassing both TD and ASD populations (Baron-Cohen et al., 2001), which would predict an intermediate position for the high AQ group, flanked by the low AQ and HFA groups. Our findings are, however, similar to those reported by Åsberg Johnels et al. (2017), who investigated the influence of AQ scores on face fixations in adults, as well as children and adolescents, and reported atypical fixations in children and adolescents with high AQ scores, but not in adults with high AQ scores. This may suggest the use of compensatory strategies in TD adults high in autistic-like traits, who may have explicitly learned to attend to the diagnostic regions of the face such as eyes and mouth (rather than do it spontaneously). It could also reflect a relatively late maturation of the social attention skills in the high AQ group, only reaching full maturation in adulthood. The current study exclusively used adult participants, which could have contributed to the absence of any differences between low and high AQ groups. In fact, in the current study the high AQ group showed a trend for looking more at the eyes than the low AQ group. In addition to the inclusion of exclusively adult participants, the median-split method to establish the high and low AQ groups could also have contributed. Comparing, for example, the upper and lower quartiles of AQ scores would ‘amplify’ differences, but our sample size did not allow this.

*HFA.* Both the HFA and mTD groups attended mostly to the eyes, but the HFA group attended a significantly smaller proportion of the time to the eyes than the mTD group. The latter would have been predicted by the hyper-arousal model (Dalton et al., 2005; Kliemann et al., 2010, 2012). However, the more specific predictions of this model, namely that the effect would be enhanced by emotionally arousing direct gaze and further by the threatening emotion of anger, were not supported. The current data shows that gaze direction and facial emotional expression did not modulate this effect. Reduced scanning of the eye region in ASD is frequently reported (Chawarska & Shic, 2009; Dalton et al., 2005; Hernandez et al., 2009; Klin et al., 2002; Pelphrey et al., 2002), though not unanimously (Bar-Haim et al., 2006; McPartland et al., 2011; Sawyer et al., 2012), which may be related to differences in stimulus characteristics and task demands (Senju & Johnson, 2009).

It should be noted that the relationship between the duration of fixation on the diagnostic areas of the face and social functioning is still not clear. Behavioural deficiencies may be observed in the absence of aberrant face scanning, while the presence of aberrant face scanning may not necessarily explain behavioural deficiencies (Sawyer et al., 2012).

Although information processing in ASD appears to be critically affected by the extent of exposure to the relevant stimuli (Clark et al., 2008; Tardif et al., 2007), if eye scanning time would be reduced in individuals with ASD, it could still be sufficient to extract the relevant social cues. Face recognition can be achieved after just one or two saccades (Hsiao & Cottrell, 2008). Although more time may be required for the perception of emotional expressions, when only basic emotions are involved emotion recognition can typically be completed in a short amount of time by HFA individuals (Wong et al., 2012). Changes in the gaze direction of the eyes were clearly discernible, as confirmed by debriefs. It is up to future research to determine the minimal fixation duration on the diagnostic face areas required to be able to perform the various social inferences necessary for efficient social functioning.

Regarding the test of the hypothesis of an autism spectrum continuum for the allocation of attention to faces, the combined analysis showed that the result for the HFA versus the TD group with high AQ scores was as predicted. The HFA group showed less scanning of the eyes and more scanning of all remaining AOIs (except for the mouth area) compared to the high AQ group. However, the HFA group did not differ in attention allocation to any of the AOIs from the TD group with low AQ, nor did the low and high AQ groups differ in any of the AOIs. The results thus only partly support the initial hypothesis of a continuum from TD with low AQ to TD with high AQ to HFA. However, the study was not specifically designed to address this question; future work should include individuals with a wider range of AQ and ADOS scores to further investigate this proposition.

### Limitations

First of all, the eye-tracking technique has some important limitations. It shows which object in the visual world is projected onto the observer’s retina, but doesn’t tell whether the object is actually processed in higher-level visual areas and perceived. It further excludes peripheral vision, which may convey important information and may affect subsequent behaviour. Last but not least, the duration fixation time is hard to interpret. Longer fixations may represent greater interest, but also greater processing difficulty. Likewise, shorter fixations may reflect lack of interest or greater processing fluency. In addition to inherent limitations in eye-tracking methodology, the current ASD participant sample was limited to adult individuals with high-functioning autism. Children and adolescents with ASD may show more diversity in their scanning patterns, while individuals with more severe forms of ASD may show distinctly different scanning patterns. The HFA group in Experiment 2 contained a significantly higher percentage of male participants than the TD groups with low and high AQ scores in Experiment 1, which could have affected the HFA versus Low/high AQ comparisons in the overall analysis. We did not screen the TD participants for the presence of close family members with ASD, and therefore cannot exclude that some may have been on the spectrum. However, none of them reported any symptoms that would indicate ASD.

## Conclusion

It was expected that the use of ecologically valid, naturally changing facial expressions would enhance any potential differences in face viewing patterns between the groups. However, TD individuals with low or high autistic-like traits did not differ from each other, while individuals with HFA differed from the matched TD group only in that they looked less at the eyes. The face scanning behaviour of all groups was independent of gaze direction and largely independent of emotional expression. Emotional expression was only influential with respect to expressions of joy, which induced enhanced exploration of the mouth region in all groups. Taken together, the results show that adults occupying distinctly different positions on the autism spectrum largely follow the same basic scanning patterns when observing dynamic faces during task-free free-viewing. The results further showed that facial expressions that were part of a dynamic, ecologically-valid sequence, reflecting changes in the emotional state of mind and attention of the depicted agent, did not induce differences in the face scanning behaviour of these individuals. Emotional face scanning seems quite robust in adults on the autism spectrum, from low AQ-TD, up to and including HFA.
